# Interhospital transfer of COVID-19 patients treated with high-flow nasal oxygen therapy

**DOI:** 10.1186/s12245-021-00385-2

**Published:** 2021-09-26

**Authors:** Elophe Dubie, François Morin, Dominique Savary, Amaury Serruys, Pascal Usseglio

**Affiliations:** 1 Emergency Department, Metropole Savoie Hospital, BP 1125, 73000 Chambery, France; 2 Emergency Department, Angers University Hospital, Angers, France; 3 IRSET (Research Institute for Environmental and Occupational Health) - UMR_S 1085, Angers University, Angers, France

**Keywords:** COVID-19, Interhospital, Transfer, High-flow, Nasal, Oxygen

## Abstract

At the start of the COVID-19 pandemic, early intubation was recommended on the basis of worldwide observations of severe hypoxemia. However, some patients were ultimately able to benefit from high-flow nasal cannula (HFNC) and thus avoid intubation. During the “second wave” (September to December 2020 in France), some emergency departments implemented HFNC in patients with severe COVID-19. The question then arose regarding the transfer of these most serious patients to intensive care units (ICU) and of the respiratory modalities to be used during this transfer. To assess the feasibility of interhospital transfers of COVID-19 patients needing HFNC, we conducted a bi-centric prospective observational study of all medical transfers of patients needing HFNC with the Chambéry and Angers (France) mobile emergency and intensive care service (SMUR) during the “second wave” of the COVID-19 pandemic in France. Analysis of these 42 patients showed no significant variation in the respiratory requirements during the transfer. Overall, 52% of patients were intubated during their stay in ICU, including three patients intubated before or during transfer. Interhospital transfer with HFNC is very high-risk, and intubation remains indicated in the most unstable patients. However, 48% of patients benefited from HFNC and were thus able to avoid intubation during their transfer and ICU stay; for these patients, intubation would probably have been indicated in the absence of available HFNC techniques.

## Introduction

At the start of the COVID-19 pandemic, early intubation was recommended on the basis of worldwide observations of severe hypoxemia. However, some patients were ultimately able to benefit from high-flow nasal cannula (HFNC) [1]. Before the COVID-19 pandemic, high-flow nasal cannula (HFNC) had been a major advance in the treatment of patients with hypoxemic lung disease, enabling them to avoid intubation and its potential complications [2, 3]. HFNC is now recommended by the French Society of Emergency Medicine in “deeply hypoxemic” COVID-19 patients to limit the use of invasive mechanical ventilation [4]. During the French “COVID-19 second wave” (September to December 2020), some emergency departments implemented HFNC in clinically severe COVID-19 patients. For some of these patients, HFNC was the only alternative to intubation, as conventional oxygen therapy was not sufficient. The question then arose regarding the transfer of these most serious patients to intensive care units (ICU) and of the respiratory modalities to be used during this transfer. To our knowledge, there are few descriptions in the literature of medical transfer of adult patients needing HFNC [5]. We aim to assess the feasibility of interhospital transfers of COVID-19 patients needing HFNC.

## Materials and methods

To assess the feasibility of interhospital transfers of COVID-19 patients needing HFNC, we compared the respiratory requirements at the start and at the end of the transfer. We conducted a bi-centric prospective observational study of all medical transfers of patients needing HFNC with the Chambéry and Angers (France) mobile emergency and intensive care service (SMUR) during the French “COVID-19 second wave”. The inclusion criterion was COVID-19 patients needing HFNC and requiring a medical interhospital transfer to an intensive care unit. The exclusion criterion was intubation or use of another mode of oxygenation than HFNC before the arrival of the medical team responsible for the transfer. We are describing here only interhospital transfers and not primary management at home by a medical team. Heater humidifiers (MR850, Fisher & Paykel) coupled to a turbine ventilator (Monnal T60, Air Liquide Medical Systems) were installed in our ambulances. The oxygen reserves in the ambulances were increased to 30 L of oxygen at 200 bars. The COVID + status of the patients was known before the treatment was started. The indication of HFNC was possible in the event of persistent polypnea and hypoxemia (based on a subjective assessment) despite conventional oxygen therapy and in the absence of immediate or foreseeable short-term intubation criteria. Biological measurement of hypoxemia was not systematic. The intubation criteria were hypercapnia (PaCO_2_ > 45 mmHg on arterial blood gas), hemodynamic instability, respiratory exhaustion, and impairment of consciousness. Initially, the flow rate was set at 50 L/min. FiO_2_ was started at 100% and adjusted down to SaO_2_ target (92–96%). The initial temperature was 37 °C and adjusted down to 34 °C if needed for better tolerance. HFNC equipment was set up at the patient’s bed. Airborne and contact protections recommended by our hospitals (respirator N95 or FFP2, gown, gloves, eye protection) were applied, as well as the renewal of the air in the ambulance by extractor (theoretical maximum flow of 700 m^3^/h). A droplet mask was placed over the patient’s nose and nasal interface, if tolerated. The number of health care professionals in contact with the patient in the ambulance had to be kept to a strict minimum. Monitoring was particularly close during different phases of transport, especially using the Rox score [6] as not to delay an intubation, for example, if the HFNC failed to maintain adequate oxygenation. Ventilation parameters (FiO_2_ and flow rate) and respiratory requirements (respiratory rate, oxygen saturation) were collected on departure and on arrival, on the patient’s medical file, and then reported in an Excel spreadsheet. We analyzed the patients' outcomes of intubation and one month survival. The study was conducted according to the reference methodology MR004 and registered in the directory of the National Institute of Health Data.

## Results

**Fig. 1:**
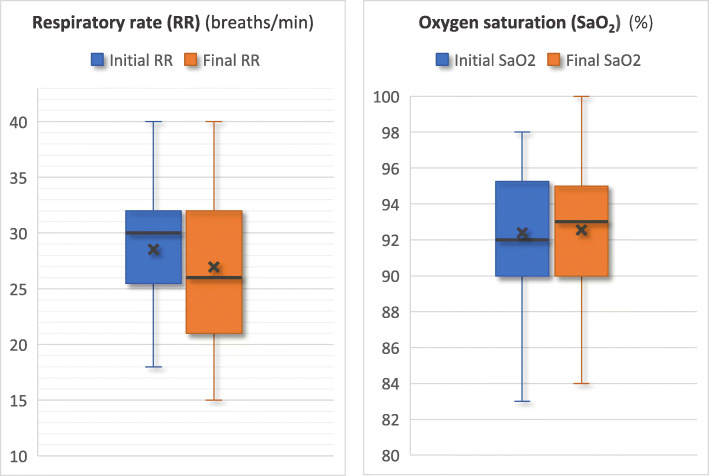
Respiratory rate and SaO_2_ at the start and end of the transfer. The horizontal line represents the median, and the *x* represents the mean. The vertical limits of the box represent the first and third quartiles. The extremities represent the maximum and minimum values

Forty-two patients were included from September 2 to December 9, 2020. The median (interquartile) age of the patients was 72 (65–78) years. A total of 69% were male. Fifty-two percent of the patients were treated for hypertension. One patient had to be intubated by the medical team in charge of the transfer before the transfer and thus was excluded from the analysis. This was the only patient excluded from the analysis. Two patients had to be intubated during the transfer by Angers SMUR. For these two patients, the respiratory rate (RR) and oxygen saturation (SaO_2_) values before intubation were entered as “final” respiratory requirements. The overall analysis of patients showed no significant variation in the respiratory requirements of the patients during the transfer (Fig. 1). At the beginning of the transfer, the RR and SaO_2_ medians were 30/min (26–32) and 92% (90–95), respectively. At intensive care unit admission, the RR and SaO_2_ were measured at 28/min (22–32) and 93% (90–95), respectively. Ventilation settings were not changed significantly during the transfer period: At the beginning of the transfer, the flow and FiO_2_ medians were 50 L/min (50–60) and 75% (53–100), respectively. At intensive care unit admission, debit and FiO_2_ were 50 L/min (50–60) and 80% (50–100), respectively. Overall, 52% of patients were intubated during their stay in the ICU, including three patients intubated before or during transfer. The median Rox score at the start of the transfer was 3.3 in patients intubated and 6.8 in non-intubated patients, after exclusion of one patient with a treatment limitation decision. No patient had to be urgently intubated upon arrival in the ICU. Survival at 1 month after admission to the ICU was 74%.

### Discussion

There are very few descriptions in the literature of medical transfer of adult COVID-19 patients needing HFNC, so our study population cannot be compared to most studies. The respiratory requirements of the patients corresponded to the indications for HFNC. However, the respiratory status of some patients was particularly severe. This may explain the high proportion of intubated patients during their ICU stay. For these patients, other causes of decompensation could have compounded the patients’ respiratory efforts, such as pulmonary embolism, heart failure, and superinfection. But in our cohort, ICU follow-up ruled out these causes of initial decompensation. When HFNC is started, the objectives are a decrease in the respiratory rate and an increase in oxygen saturation. Then, during mobilizations and ambulance transport, the objective is to maintain the stability of these respiratory requirements. Between the patient’s room and the intensive care ambulance, during which the power supply to the heater humidifier is interrupted, humidification and heating of the air-oxygen mixture are no longer guaranteed. Did this lack of warming and humidification contribute to the sudden deterioration of the respiratory state of the two patients who were intubated? This led us to recommend that the need for a prolonged interruption of power supply would contraindicate a transfer with HFNC, unless the transfer is after a prior weaning trial. Interhospital transfer with HFNC is very high-risk, and intubation remains indicated in the most unstable patients.

However, 48% of patients benefited from HFNC and were thus able to avoid intubation during their transfer and ICU stay; for these patients, intubation would probably have been indicated in the absence of available HFNC techniques. Ideally, we would have liked to compare the survival of two cohorts of patients. Are these patients (who are transferred on HFNC) having overall better survival rates or approximately the same as their intubated cohorts? The main obstacle to using HFNC in COVID-19 patients was the fear of an increased risk of contamination for personnel due to the aerosolization produced when using HFNC. However, a few trials did not appear to show that there is an increased risk of contamination with HFNC compared with other modes of oxygenation, including high concentration masks [7–9]. Our small trial needs to be expanded to a larger patient population for more concrete evidence of HFNC benefits in interhospital transfers. Finally, alternatives to HFNC are possible, such as non-invasive ventilation or continuous positive airway pressure.

## Conclusions

This study shows the feasibility of interhospital transfers of COVID-19 patients needing HFNC. No significant variation in the respiratory requirements of the patients was observed during the transfer. However, these transfers with HFNC are very high-risk.

## Data Availability

The datasets analyzed during the current study are available from the corresponding author on reasonable request.
